# Vitamin K: an old vitamin in a new perspective

**DOI:** 10.4161/19381972.2014.968490

**Published:** 2015-01-21

**Authors:** U Gröber, J Reichrath, MF Holick, K Kisters

**Affiliations:** 1 Academy for Micronutrient Medicine; Essen, Germany; 2 Saarland University Clinics; Homburg/Saar, Germany; 3 Boston University Medical Center; Boston, MA USA; 4 St. Anna Hospital; Medical Clinic 1; Herne, Germany

**Keywords:** bone health, cardiovascular health, matrix GLA protein, menaquinone-7, osteocalcin, phylloquinone, vitamin K

## Abstract

The topic of “Vitamin K” is currently booming on the health products market. Vitamin K is known to be important for blood coagulation. Current research increasingly indicates that the antihaemorrhagic vitamin has a considerable benefit in the prevention and treatment of bone and vascular disease. Vitamin K_1_ (phylloquinone) is more abundant in foods but less bioactive than the vitamin K_2_ menaquinones (especially MK-7, menaquinone-7). Vitamin K compounds undergo oxidation-reduction cycling within the endoplasmic reticulum membrane, donating electrons to activate specific proteins via enzymatic gamma-carboxylation of glutamate groups before being enzymatically reduced. Along with coagulation factors (II, VII, IX, X, and prothrombin), protein C and protein S, osteocalcin (OC), matrix Gla protein (MGP), periostin, Gas6, and other vitamin K-dependent (VKD) proteins support calcium homeostasis, inhibit vessel wall calcification, support endothelial integrity, facilitate bone mineralization, are involved in tissue renewal and cell growth control, and have numerous other effects. The following review describes the history of vitamin K, the physiological significance of the K vitamers, updates skeletal and cardiovascular benefits and important interactions with drugs.

## Vitamin K: A Review of its History

The discovery of vitamin K can be traced back to the research of Carl Peter Henrik Dam at the Biochemical Institute of the University of Copenhagen from 1928 to 1930. In his work on cholesterol metabolism, the Danish biochemist observed a spontaneous tendency to hemorrhage in chicks fed for longer than 2 to 3 weeks on cholesterol- and fat-free chicken feed. This coagulation disorder was combined with a lowered prothrombin content (prothrombin = factor II) of the blood.^1-3^ At that time, as none of the hitherto known vitamins (e.g. vitamins A, C and D) were capable of preventing the coagulation disorder, Dam postulated a new, fat-soluble vitamin, which regulates coagulation. The latter was apparently present in green vegetables and liver, as supplementary feeding with these nutrients resulted in normal blood coagulation in the animals. Moreover, Dam successfully treated the chickens’ hemorrhages with an ether extract obtained from lucerne (alfalfa). Dam called the antihaemorrhagic vitamin “vitamin K” (after “Koagulation:" coagulation).^4^

In the 1930s, several working groups researched the isolation and identification of vitamin K. At this time, a US American research group working with the biochemist Edward Albert Doisy succeeded in isolating the antihaemorrhagic vitamin K and elucidating its chemical naphthoquinone ring structure. In 1943, the 2 researchers, Dam und Doisy, were jointly awarded the Nobel prize for medicine for the discovery and elucidation of the chemical structure of vitamin K.^5,6^

The precise biochemical function of vitamin K was not finally resolved until the end of the 1970s. As a coenzyme, vitamin K is essential for the γ-carboxylation of specific glutamic acid (Glu) residues in a number of vitamin K-dependent proteins. The resultant γ-carboxyglutamic acid (Gla) compounds can effect complex binding of calcium ions, leading to a protein conformational change, which is a precondition for its physiological function. In this way, e.g., by means of posttranslational modification, the clotting factors II (prothrombin), VII, IX and X develop from precursors.^7-9^

**Figure 1.**
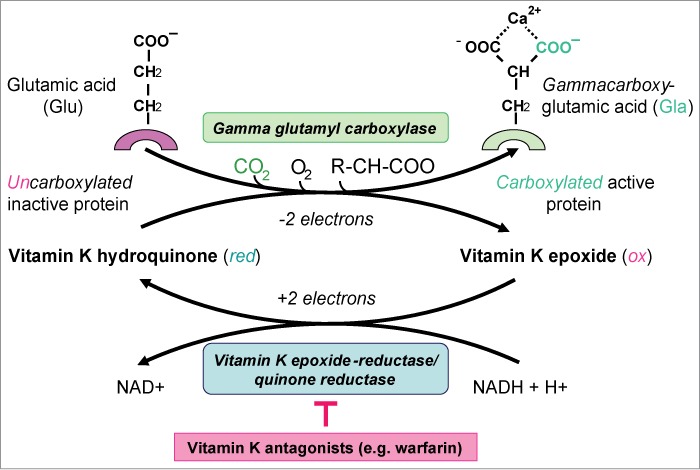
In the vitamin K cycle, vitamin K-dependent gamma-carboxyglutamic acid (Gla) proteins are carboxylated and activated.

In this context, the significance of the vitamin K cycle was also recognized: γ-carboxylation is catalyzed by a microsomal carboxylase and requires CO_2_ and molecular oxygen. Vitamin K hydroquinone is required as a cofactor. The oxidation of the hydroquinone to vitamin K 2,3-epoxide supplies the energy required for the abstraction of a proton of the gamma carbon of the glutamic acid (Glu) residue, resulting in a carbanion, which is then carboxylated to γ-carboxyglutamic acid (Gla) (**Fig. 1**). Vitamin K 2,3-epoxide is subsequently regenerated to vitamin K hydroquinone by the enzymes vitamin K epoxide and quinone reductase.^10-12^ The γ-carboxylation is thus characterized by a cyclical transformation, in which oxidised and reduced forms of vitamin K are involved as the driving factors. The inhibition of these 2 enzymes by vitamin K antagonists, such as phenprocoumon and warfarin, has considerable medical significance, which is utilised in anticoagulation therapy.^13^ Currently, approximately 14 vitamin K-dependent proteins are known, with broad spectrum efficacy on haemostasis, calcium metabolism, control of cell growth, apoptosis and signal transduction (**Table 1**).^14-17^ Following elucidation of the vitamin's haemostasiological significance and research into further vitamin K-dependent Gla proteins, such as osteocalcin (BGP = bone Gla protein) and matrix Gla protein (MGP), current research is focusing on the vitamin's effect on bone and vascular health.^10,13,18^

**Table 1.** Vitamin K-dependent gamma carboxyglutamate (Gla) proteins (e.g., prothrombin, osteocalcin)

Vitamin K dependent Gla protein	Function
Liver	Hepatic carboxylation
Clotting factors II, VII, X and XII	Haemostasis (procoagulant activity)
Protein C, S and Z	Haemostasi (anticoagulant activity)
Various tissues	Extra hepatic carboxylation
Osteocalcin	Calcium and bone metabolism
Matrix-Gla-Protein	Inhibitor of vascular calcification (cartilaginous tissue, vascular wall of the vascular smooth muscle cells)
Growth-arrest specific gene 6 (Gas6)	Cell growth (endothelium, smooth muscle cells), apoptosis, phagocytosis (?)
Transmembrane GLA-protein	Signal transduction to phosphatidylserine (?)
Periostin	Bone metabolism, cell migration, angiogenesis (?)
Other: carboxylase, transthyretin, Gla-rich-Protein (GRP)	To date mainly unknown

## The K Vitamers: Nomenclature, Structure and Occurrence

Vitamin K is not a single unified substance, but rather a group of closely related derivatives with a 2-methyl-1,4-naphthoquinone structure as a common framework. All vitamin K derivatives contain this framework, which is also called menadione. Menadione is not a naturally occurring substance, but it can be manufactured synthetically and it is also known as *vitamin K_3_*. The individual substances from the K vitamin group are also known as K vitamers. They differ from each other mainly with regard to the length and saturation of the isoprenoid side chain at C3.^16^

The most important naturally occurring K vitamins are phylloquinone (2-methyl-3-phytyl-1,4-naphthoquinone, phytomenadione), which is contained in green plants and also known as *vitamin K_1_*, and menaquinone, with side chains of varying length, which is formed from intestinal bacteria (e.g., Bacteroides) and is also known as *vitamin K_2_*. Vitamin K_1_ contains a phytyl side chain with 20 C atoms, i.e. a monounsaturated, lipophilic side chain with 4 isoprene units. In plants, for example, phylloquinone is a functional and structural component of photosynthesis. Vitamin K_1_ is synthesized by plants and algae. It thus occurs mainly in green leafy vegetables, such as kale (145 μg/100 g), Brussels sprouts (177 μg/100 g), broccoli (180 μg/100 g) and spinach (380 μg/100 g), accounting for approximately 90% of the vitamin K ingested with the diet. In addition, vitamin K is found in some vegetable oils (e.g., soya oil: 193 μg/100 g, rapeseed oil: 127 μg/100 g) and in foods of animal origin (e.g., liver: 5 μg/100 g and eggs: 2 μg/100 g).^19-21^

**Figure 2.**
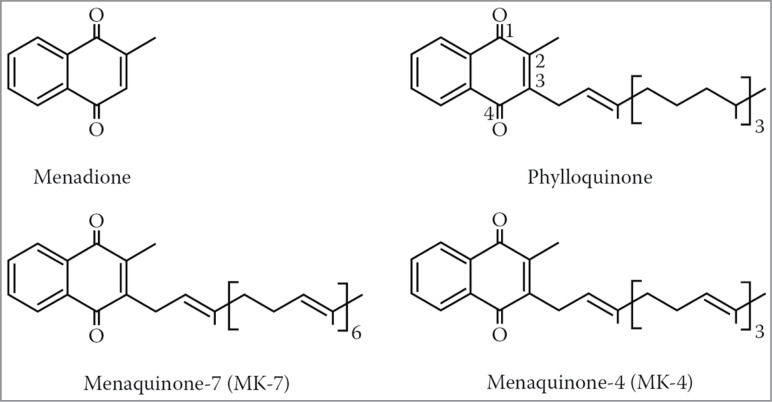
Structural formulae of biologically active K vitamers.

*Vitamin K_2_* consists of a group of menaquinones, which are characterized by the length of their isoprenoid side chain, a lipophilic, polyunsaturated side chain of variable length (**Fig. 2**). A menaquinone with 7 isoprenoid units was formerly called vitamin K_35_, as one isoprenoid unit contains 5 C atoms. Today, menaquinones are generally called MK-n, where n signifies the number of isoprenoid units. With regard to preventive and therapeutic aspects, menaquinone-4 (MK-4) and menaquinone-7 (MK-7) are among the most important forms of vitamin K_2_ with 4 and 7 isoprenoid units, respectively.^16,19,22^ Menaquinones are mainly found in foods of animal origin, such as bovine liver and in bacterially fermented foods, such as yoghurt and some types of cheese (e.g., MK-8 and MK-9: 5–20 μg/100 g). The richest source of MK-7 at 10 μg/g is a Japanese dish called natto, which has a long nutritional tradition and is made from bacterially fermented soya beans. The bacterium that produces MK-7 in soya is called *Bacillus subtilis natto*. The earliest written documentation on natto can be found in the Japanese book “Shin Sarugakki” by Fujiwara no Akihira, who lived from 989–1066 BC.^23-25^

## The Vitamin K Requirement

There are no precise recommendations for the vitamin K requirement and those of the nutritional associations are usually based on the hepatic requirement for the formation of blood clotting factors. Taking the plasma thrombin level into consideration, a daily vitamin K intake of 1 μg per kg body weight is recommended as being adequate for all age groups beyond the neonatal phase.

In a recent study by the University of Maastricht, however, in which 896 blood samples from healthy subjects were analyzed, it was shown that, although all coagulation proteins were completely carboxylated by vitamin K, a high concentration of uncarboxylated Gla proteins (osteocalcin, matrix Gla protein) was present in the majority of the subjects investigated. Uncarboxylated osteocalcin (ucOC) and uncarboxylated matrix Gla protein (ucMGP) are functional laboratory parameters for a vitamin K deficiency and are associated with an increased risk of bone fractures and vascular complications. Based on the results of this study, it must be assumed that the majority of the population has an inadequate supply of vitamin K.,^26^

## Effect of Vitamin K on the Bones and Vascular System

As a result of vitamin K-mediated γ-carboxylation, the various Gla proteins can bind calcium ions and are activated in this way. Carboxylated osteocalcin (cOC) binds calcium in the bone tissue, which is incorporated into the hydroxylapatite of the bone with the help of the osteoblasts. A low dietary vitamin K intake and high proportion of uncarboxylated osteocalcin (ucOC) are independent risk factors for hip fractures.^27-30^

The production and activation of osteocalcin (OC) is regulated by vitamin K and 1,25-dihydroxyvitamin D [1,25(OH)_2_D; calcitriol].^30,31^ 1,25(OH)_2_D promotes the transcription of the osteocalcin gene, whereas vitamin K promotes the posttranscriptional carboxylation of Gla residues in the osteocalcin propeptide.^31,32^ Furthermore it was demonstrated that 1,25(OH)_2_D enhances the activity of γ-glutamyl carboxylase, suggesting that the carboxylation of osteocalcin is stimulated by vitamin D and that menaquinone-4 stimulates 1,25-dihydroxyvitamin D3-induced mineralization by human osteoblasts.^33^ There is growing evidence about the synergistic effect on bone health of vitamin K and vitamin D. But further data is required in order to have a complete understanding of the complex interaction between vitamin K, vitamin D and bone metabolism.

**Figure 3.**
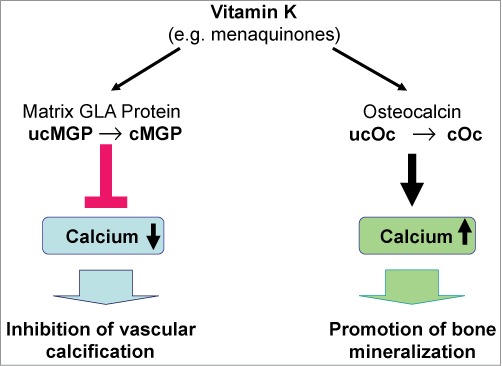
Effect of vitamin K on bone and vascular health.

Whereas carboxylated osteocalcin (cOC) promotes the incorporation of calcium into the bone matrix, thus supporting bone metabolism, the vitamin K-dependent matrix Gla protein (cMGP) counteracts vascular calcification and age-related wear and tear on the arteries and protects the blood vessels from calcium overload (**Fig. 3**).^34,35^ There are increasing indications that normal dietary intake of the vitamin K allowance recommended by the nutritional associations is insufficient for the γ-carboxylation of osteocalcin and matrix Gla protein.^26^

**Figure 4.**
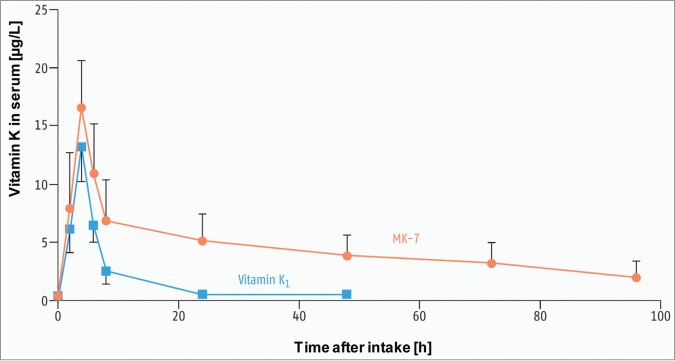
Comparison of oral bioavailability of vitamin K1 and MK-7: vitamin K serum levels following a single dose of 1 mg vitamin K1 or 1 mg MK-7.^49^

**Figure 5.**
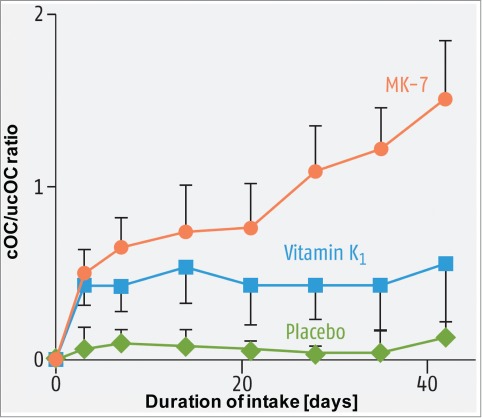
Carboxylation of osteocalcin by vitamin K1 and MK-7: Vitamin K1 or MK-7 were supplemented daily at a dose of 0.22 μmol in the form of tablets or capsules. Initially, the ratio between carboxylated (cOC) and uncarboxylated osteocalcin (ucOC) was 1.74 in the MK-7 group, 1.8 in the vitamin K1 group and 1.7 in the placebo group. In the placebo group, only vitamin K1 was determined. After approximately 3 days, vitamin K1 and MK-7 increased the carboxylation of osteocalcin, but only the intake of MK-7 led to a further increase in the degree of carboxylation.^49^

### Bone health

In the Nurses’ Health Study, which investigated 72,327 women aged from 38–63 years, the effect of daily vitamin K intake on bone fragility was investigated over a 10-year period. It was shown that women with a daily vitamin K intake of ≥ 109 μg had a 30% reduction in the risk of hip fracture compared to women with an intake of <109 μg (RR: 0.70; 95% CI: 0.53, 0.93).^28^ In a double-blind, placebo-controlled study with 55 adolescents, the proportion of uncarboxylated osteocalcin (ucOC) was significantly reduced compared to placebo by a daily supplement of 45 μg vitamin K_2_ as menaquinone-7 and the proportion of carboxylated osteocalcin (cOC) was increased, indicating improved bone mineralization.^36^

A meta-analysis of 13 randomized controlled studies investigated the effect of vitamin K supplementation as vitamin K_1_ (1–10 mg daily) or vitamin K_2_ (15–45 mg MK-4 daily) on the fracture rate and bone density. It was shown that, compared with placebo, particularly vitamin K_2_ as MK-4 reduces the risk of vertebral fractures by 60% (OR: 0.40; 95% CI, 0.25–0.65), of hip fractures by 77% (OR: 0.23; (95% CI, 0.12–0.47) and of non-vertebral fractures by 81% (OR: 0.19; 95% CI, 0.15–0.35).^37^ In a recent 3-year placebo-controlled study in 244 healthy postmenopausal women, a daily supplement of 180 μg vitamin K_2_ as MK-7 led to a significant improvement in bone density, bone health and bone strength. The quotient of ucOC/cOC served as a marker for the vitamin K status and was considerably improved by MK-7.^38^

Increased levels of ucOC are also found in patients with fractures during treatment with amino-bisphosphonates. In a randomized study in 241 postmenopausal women, a supplement of 45 mg vitamin K_2_ (MK-4, menaquinone-4) over a period of 24 months led to a significant rise in carboxylated osteocalcin (cOC) and a significantly reduced fracture rate compared with the control group. The osseous efficacy of the bisphosphonates (e.g., risedronate) used in osteoporosis therapy can be improved by concurrent supplementation with vitamin K_2_ (MK-4: 45–60 mg/d), which has been confirmed by the results of clinical studies.^39-42^

### Vascular health

In the Rotterdam Study, a large-scale, population-based study with 4,807 Dutch women and men (age: 55+), the effect of dietary vitamin K_1_ and vitamin K_2_ over a 10-year period (1990 to 2000) was investigated with regard to the risk of coronary heart disease, arterial calcification and overall mortality. This study found that vitamin K_1_ (intake: ∼250 μg/day) had no protective effect on the cardiovascular system or overall mortality. Vitamin K_2_ (intake: ∼25 μg/day) reduced the relative risk of dying of heart disease by 57%. Vitamin K_2_ also markedly reduced the occurrence of coronary heart disease (by 41%) and overall mortality (by 36%). Vitamin K_2_ even reduced the risk of severe arterial calcification by 52% (OR: 0.48).^43^

Carboxylated MGP is an important inhibitor of vascular calcification. Accordingly, uncarboxylated MGP (ucMGP) is an independent risk factor for arteriosclerosis. In a recent placebo-controlled study, a daily supplement of 180 μg or 360 μg MK-7 led to a significant reduction in uncarboxylated MGP (ucMGP) of 31% and 46%, respectively, compared with placebo.^44^ Due to premature vascular calcification, dialysis patients have a higher cardiovascular risk. In those affected, elevated levels of uncarboxylated MGP are frequently present, indicating insufficient dietary intake of vitamin K. In a recent study with dialysis patients, supplementation with 360 μg, 720 μg or 1080 μg MK-7 3 times weekly over an 8-week period significantly reduced the proportion of inactive MGP by 17%, 33% and 46%, respectively.^45,46^ It must therefore be assumed that vitamin K_2_ supplementation can improve the individual cardiovascular risk in cardiovascular and dialysis patients.

In addition, the protective effect of vitamin K_2_ in vascular disease makes it of interest for patients with diabetes mellitus. In patients with diabetes, elevated levels of uncarboxylated MGP are also associated with an increased risk of vascular calcification. Furthermore, a recent placebo-controlled study in 42 healthy men showed that supplementation with 30 mg vitamin K_2_ as MK-4 (3× daily) improved the insulin production and insulin sensitivity of the cells through activation of osteocalcin (ucOC → cOC), compared with placebo. Carboxylated osteocalcin (cOC) appears to be an endogenous hormone, which also improves insulin metabolism.^45,46^ Also of interest are the results of animal studies, which showed that accumulation of vitamin K_2_ (MK-4) in the arterial wall was 3 times higher than that of vitamin K_1_. In this context, arterial calcification triggered by warfarin was also completely prevented by vitamin K_2_ but not by vitamin K_1_. As a result of its isoprenoid-rich structure, vitamin K_2_ also appears to have a favorable effect on cholesterol values.^47^

### Further therapeutic indications

Due to its anti-inflammatory, anti-oxidative and anticarcinogenic properties, vitamin K, particularly MK-7, may be of interest in a number of other diseases (e.g., cancer, diabetes, age-related macular degeneration [AMD]); over the next few years, studies will show whether this is the case. Furthermore, because of its structural similarity to coenzyme Q10, it is likely that MK-7 is a Q10 mimetic with respect to the mitochondria and supports mitochondrial adenosine triphosphate (ATP) production in the respiratory chain.

## K Vitamers: Differences in Efficacy

Of the various K vitamers, i.e., phylloquinone and vitamin K_2_ as menaquinone-4 (MK-4) or menaquinone-7 (MK-7), vitamin K_1_ and menaquinone-7 are of most interest as food supplements. MK-7, which is obtained from natto, shows some physico-chemical advantages compared with vitamin K_1_. Due to its molecular structure, menaquinone-7 (MK-7) is more lipophilic and has a much longer half-life (3 days) than vitamin K_1_. Regular MK-7 intake therefore results in blood levels that are not only more stable but also approximately 7–8 times higher. Compared with vitamin K_1_, distribution of MK-7 in the various tissues is significantly better. MK-7 is thus more efficient in the carboxylation of extrahepatic (e.g., osteocalcin) and hepatic (e.g., prothrombin) proteins (**Figs. 4, 5**).^48,49^ Compared with MK-7, no oral bioavailability has been determined for MK-4 at nutritional doses (e.g., 420 μg MK-4). Therefore, the small quantities of MK-4 contained in the diet do not contribute to the buildup of vitamin K status or to the degree of carboxylation of vitamin K-dependent proteins.^50^

## Interaction with Vitamin K Antagonists

As a result of the considerably better bioavailability of MK-7, the risk of a pharmacodynamic interaction with vitamin K antagonists is also markedly higher than with vitamin K_1_. Whereas studies showed a reduction in the International Normalized Ratio (INR) value (from 2 to 1.5) at an intake of >300 μg vitamin K_1_ daily, this occurred at >100 μg with MK-7. Many nutritional supplements are currently marketed at a daily dosage of 45 μg and over. Recent dose-finding studies at the University of Maastricht, which investigated the effect of 10 μg, 20 μg and 45 μg MK-7 daily on the anticoagulant properties of vitamin K antagonists, showed that even with a daily supplement of < 10 μg MK-7, a significant disturbance of blood coagulation control can occur. Dr Theuwissen's working group therefore advises against MK-7 supplements in patients undergoing treatment with vitamin K antagonists.^48,51^

## Summary for Clinical Practice

Vitamin K–particularly MK-7–is currently enjoying a genuine boom in the health products branch, comparable with the vitamin D boom of around 3 years ago. For preventive purposes, a recommended daily supplement of 0.5–1.0 μg MK-7 per kg body weight is an acceptable guideline. In the treatment of diseases such as osteoporosis, the equivalent daily dose would be 2–4 μg per kg body weight. Medical and pharmaceutical practitioners should be conversant with the basic aspects and particular features of the K vitamers in order to offer patients competent advice. Whereas recent studies show that vitamin K_2_ is gaining importance in the prevention and therapy of bone and vascular disease, its high interaction potential with anticoagulants remains a problem!

## References

[1] Dam H Cholesterinstoffwechsel in Hühnereiern und Hühnchen. Biochem Z 1929; 215:475-92

[2] Dam H Haemorrhages in chicks reared on artificial diets: a new deficiency disease. Nature 1934; 133:909-10; http://dx.doi.org/10.1038/133909b0

[3] Dam H The antihaemorrhagic vitamin of the chick. Occurrence and chemical nature. Nature 1935; 135:652-3; http://dx.doi.org/10.1038/135652b0

[4] Dam H, Schonheyder F, Tage-Hansen E. Studies on the mode of action of vitamin K. Biochem J 1936; 30:1075-9; PMID:16746121 16746121 10.1042/bj0301075 PMC1263146

[5] McKee RW, Binkley SB, MacCorquodale DW, Thayer SA, Doisy EA The isolation of vitamins K1 and K2. J Am Chem Soc 1939; 61:1295; http://dx.doi.org/10.1021/ja01874a507

[6] Thayer SA, McKee RW, Binkley SB, MacCorquodale DW, Doisy EA The assay of vitamins K1 and K2. Proc Soc Exp Biol Med 1939; 41:194-7

[7] Nelsestuen GL, Suttie JW. Mode of action of vitamin K. calcium binding properties of bovine prothrombin. Biochemistry 1972; 11(26): 4961-4; PMID:4118102; http://dx.doi.org/10.1021/bi00776a013 4118102

[8] Stenflo J, Suttie JW. Vitamin K-dependent formation of gamma-carboxyglutamic acid. Annu Rev Biochem 1977; 46:157-72; PMID:332061; http://dx.doi.org/10.1146/annurev.bi.46.070177.001105 332061

[9] Esmon CT, Suttie JW. Vitamin K-dependent carboxylase: solubilization and properties. J Biol Chem 1976; 251(20): 6238-43; PMID:977568 977568

[10] Dowd P, Hershline R, Ham SW, Naganathan S. Vitamin K and energy transduction: a base strength amplification mechanism. Science 1995; 269(5231): 1684-91; PMID:7569894; http://dx.doi.org/10.1126/science.7569894 7569894

[11] Berkner KL. The vitamin K-dependent carboxylase. Annu Rev Nutr 2005; 25:127-49; PMID:16011462; http://dx.doi.org/10.1146/annurev.nutr.25.050304.092713 16011462

[12] Suttie JW. Synthesis of vitamin K-dependent proteins. FASEB J 1993; 7(5): 445-452; PMID:8462786 8462786 10.1096/fasebj.7.5.8462786

[13] Bell RG. Metabolism of vitamin K and prothrombin synthesis: anticoagulants and the vitamin K-epoxide cycle. Fed Proc 1978; 37(12): 2599-604; PMID:359368 359368

[14] Lian JB, Friedman PA. The vitamin K-dependent synthesis of gamma-carboxyglutamic acid by bone microsomes. J Biol Chem 1978; 253(19): 6623-6; PMID:567642 567642

[15] Suttie JW. Mechanism of action of vitamin K: synthesis of gamma-carboxyglutamic acid. CRC Crit Rev Biochem 1980; 8(2):191-223; PMID:6772376; http://dx.doi.org/10.3109/10409238009105469 6772376

[16] Suttie JW. Vitamin K: In Health and Disease. CRC Press, 2009

[17] Rishavy MA, Berkner KL. Insight into the coupling mechanism of the vitamin K-dependent carboxylase: mutation of histidine 160 disrupts glutamic acid carbanion formation and efficient coupling of vitamin K epoxidation to glutamic acid carboxylation. Biochemistry 2008; 47(37): 9836-46; PMID:18717596; http://dx.doi.org/10.1021/bi800296r 18717596 PMC3293503

[18] Shearer MJ, Bach A, Kohlmeier M. Chemistry, nutritional sources, tissue distribution and metabolism of vitamin K with special reference to bone health. J Nutr 1996; 126(4 Suppl): 1181S-6S; PMID:8642453 8642453 10.1093/jn/126.suppl_4.1181S

[19] Booth SL, Suttie JW. Dietary intake and adequacy of vitamin K. J Nutr 1998; 128(5): 785-8; PMID:9566982 9566982 10.1093/jn/128.5.785

[20] Suttie JW. The importance of menaquinones in human nutrition. Annu Rev Nutr 1995; 15:399-417; PMID:8527227; http://dx.doi.org/10.1146/annurev.nu.15.070195.002151 8527227

[21] Schurgers LJ, Vermeer C. Determination of phylloquinone and menaquinones in food. Effect of food matrix on circulating vitamin K concentrations. Haemostasis 2000; 30(6):298-307; PMID:11356998 11356998 10.1159/000054147

[22] Olson RE. The function and metabolism of vitamin K. Annu Rev Nutr 1984; 4:281-337; PMID:6380538; http://dx.doi.org/10.1146/annurev.nu.04.070184.001433 6380538

[23] Shearer MJ, Newman P. Metabolism and cell biology of vitamin K. Thromb Haemost 2008; 100:530-47; PMID:18841274 18841274

[24] History of miso, soybean jiang (China), jang (Korea) and tauco/taotjo (Indonesia) (200 BC-2009): Extensively annotated bibliography and sourcebook. Compiled by Shutleff W, Aoyagi A. Soyinfo Center, Lafayette, 2009

[25] Shearer M. Vitamin K. Lancet 1995, 345(8944): 229-34; PMID:7823718 7823718 10.1016/s0140-6736(95)90227-9

[26] Theuwissen E, Magdeleyns EJ, Braam LA. Vitamin K-status in healthy volunteers. Food Funct 2014; 5(2): 229-34; PMID:24296867; http://dx.doi.org/10.1039/c3fo60464k 24296867

[27] Szulc P, Chapuy M-C, Meunier PJ, Delmas PD. Serum undercarboxylated osteocalcin is a marker of the risk of hip fracture in elderly women. J Clin Invest 1993; 91(4): 1769-74; PMID:8473517; http://dx.doi.org/10.1172/JCI116387 8473517 PMC288157

[28] Feskanich D, Weber P, Willett WC, Rockett H, Booth SL, Colditz GA. Vitamin K intake and hip fractures in women: a prospective study. Am J Clin Nutr 1999; 69(1): 74-9; PMID:9925126 9925126 10.1093/ajcn/69.1.74

[29] Yamauchi M, Yamaguchi T, Nawata K, Takaoka S, Sugimoto T. Relationships between undercarboxylated osteocalcin and vitamin K intakes, bone turnover, and bone mineral density in healthy women. Clin Nutr 2010; 29(6): 761-5; PMID:20332058; http://dx.doi.org/10.1016/j.clnu.2010.02.010 20332058

[30] Price PA. Role of vitamin-K-dependent proteins in bone metabolism. Annu Rev Nutr, 1988; 8:565-83; PMID:3060178; http://dx.doi.org/10.1146/annurev.nu.08.070188.003025 3060178

[31] Furie B, Furie BC. Molecular basis of vitamin K-dependent gamma-carboxylation. Blood, 1990; 75(9): 1753-62; PMID:2184900 2184900

[32] Arbour NC, Darwish HM, DeLuca HF, Transcriptional control of the osteocalcin gene by 1,25-dihydroxyvitamin D-2 and its 24-epimer in rat osteosarcoma cells. Biochim Biophys Acta, 1995; 1263(2): 147-53; PMID:7640305; http://dx.doi.org/10.1016/0167-4781(95)00091-T 7640305

[33] Koshihara Y, Hoshi K, Ishibashi H, Shiraki M. Vitamin K2 promotes 1alpha, 25(OH)2 vitamin D3-induced mineralization in human periosteal osteoblasts. Calcif Tissue Int 1996; 59(6): 466-73; PMID:8939773 8939773 10.1007/BF00369212

[34] Braam LA, Hoeks AP, Brouns F, Hamulyák K, Gerichhausen MJ, Vermeer C. Beneficial effects of vitamin K on the elastic properties of the vessel wall in postmenopausal women: a follow-up study. Thromb Haemost 2004; 91(2): 373-80; PMID:14961167 14961167 10.1160/TH03-07-0423

[35] Vermeer C, Shearer MJ, Zittermann A, Bolton-Smith C, Szulc P, Hodges S, Walter P, Rambeck W, Stöcklin E, Weber P. Beyond deficiency: potential benefits of increased intakes of vitamin K for bone and vascular health. Eur J Nutr 2004; 43(6):1-11; PMID:15309455; http://dx.doi.org/10.1007/s00394-004-0480-4 15309455

[36] van Summeren MJ, Braam LA, Lilien MR, Schurgers LJ, Kuis W, Vermeer C. The effect of menaquinone-7 (vitamin K2) supplementation on osteocalcin carboxylation in healthy prepubertal children. Br J Nutr 2009; 102(8): 1171-8; PMID:19450370; http://dx.doi.org/10.1017/S0007114509382100 19450370

[37] Cockayne S, Adamson J, Lanham-New S, Shearer MJ, Gilbody S, Torgerson DJ. Vitamin K and the prevention of fractures: systematic review and meta-analysis of randomized controlled trials. Arch Intern Med 2006; 166(12): 1256-61; PMID:16801507; http://dx.doi.org/10.1001/archinte.166.12.1256 16801507

[38] Knapen MH, Drummen NE, Smit E, Vermeer C, Theuwissen E. Three-year low-dose menaquinone-7 supplementation helps decrease bone loss in healthy postmenopausal women. Osteoporos Int 2013; 24(9): 2499-507; PMID:23525894; http://dx.doi.org/10.1007/s00198-013-2325-6 23525894

[39] Aonuma H, Miyakoshi N, Hongo M, Kasukawa Y, Shimada Y. Low serum levels of undercarboxylated osteocalcin in postmenopausal osteoporotic women receiving an inhibitor of bone resorption. Tohoku J Exp Med 2009; 218(3): 201-5; PMID:19561390; http://dx.doi.org/10.1620/tjem.218.201 19561390

[40] Hirao M, Hashimoto J, Ando W, Ono T, Yoshikawa H. Response of serum carboxylated and undercarboxylated Osteocalcin to alendronate monotherapy and combined therapy with vitamin K2 in postmenopausal women. J Bone Miner Metab 2008; 26 (3): 260-4; PMID:18470667; http://dx.doi.org/10.1007/s00774-007-0823-3 18470667

[41] Matsumoto Y, Mikuni-Takagaki Y, Kozai Y, Miyagawa K, Naruse K, Wakao H, Kawamata R, Kashima I, Sakurai T. Prior treatment with vitamin K(2) significantly improves the efficacy of risedronate. Osteoporos Int 2009; 20(11): 1863-72; PMID:19280272; http://dx.doi.org/10.1007/s00198-009-0888-z 19280272 PMC2765650

[42] Shiraki M, Yamazaki Y, Shiraki Y, Hosoi T, Tsugawa N, Okano T. High level of serum undercarboxylated osteocalcin in patients with incident fractures during bisphosphonate treatment. J Bone Miner Metab 2010; 28(5): 578-84; PMID:20221651; http://dx.doi.org/10.1007/s00774-010-0167-2 20221651

[43] Geleijnse JM, Vermeer C, Grobbee DE, Schurgers LJ, Knapen MH, van der Meer IM, Hofman A, Witteman JC. Dietary intake of menaquinone is associated with a reduced risk of coronary heart disease: the Rotterdam Study. J Nutr, 2004; 134(11): 3100-5; PMID:15514282 15514282 10.1093/jn/134.11.3100

[44] Dalmeijer GW, van der Schouw YT, Magdeleyns E, Ahmed N, Vermeer C, Beulens JW. The effect of menaquinone-7 supplementation on circulating species of matrix Gla protein. Atherosclerosis 2012; 225(2):397-402; PMID:23062766; http://dx.doi.org/10.1016/j.atherosclerosis.2012.09.019 23062766

[45] Caluwé R, Vandecasteele S, Van Vlem B, Vermeer C, De Vriese AS. Vitamin K2 supplementation in haemodialysis patients: a randomized dose-finding study. Nephrol Dial Transplant 2013; 29(7):1385-90; PMID:24285428; http://dx.doi.org/10.1093/ndt/gft464 24285428

[46] Cheung CL, Sahni S, Cheung BM, Sing CW, Wong IC., Vitamin K intake and mortality in people with chronic kidney disease from NHANES III. Clin Nutr 2014; pii: S0261-5614(14):00086-7; PMID:24745600; http://dx.doi.org/10.1016/j.clnu.2014.03.011 24745600

[47] Spronk HM, Soute BA, Schurgers LJ, Thijssen HH, De Mey JG, Vermeer C. Tissue-specific utilization of menaquinone-4 results in the prevention of arterial calcification in warfarin-treated rats. J Vasc Res 2003; 40(6): 531-7; PMID:14654717; http://dx.doi.org/10.1159/000075344 14654717

[48] Sato T, Schurgers LJ, Uenishi K. Comparison of menaquinone-4 and menaquinone-7 bioavailability in healthy women. Nutr J 2012; 11:93; PMID:23140417; http://dx.doi.org/10.1186/1475-2891-11-93 23140417 PMC3502319

[49] Schurgers LJ, Teunissen KJ, Hamulyák K, Knapen MH, Vik H, Vermeer C. Vitamin K-containing dietary supplements: comparison of synthetic vitamin K1 and natto-derived menaquinone-7. Blood 2007; 109(8): 3279-83; PMID:17158229; http://dx.doi.org/10.1182/blood-2006-08-040709 17158229

[50] Sato T, Schurgers LJ, Uenishi K. Comparison of menaquinone-4 and menaquinone-7 bioavailability in healthy women. Nutr J 2012; 11:93; PMID:23140417; http://dx.doi.org/10.1186/1475-2891-11-93 23140417 PMC3502319

[51] Theuwissen E, Teunissen KJ, Spronk HM, Hamulyák K, Ten Cate H, Shearer MJ, Vermeer C, Schurgers LJ. Effect of low-dose supplements of menaquinone-7 (vitamin K2) on the stability of oral anticoagulant treatment: dose-response relationship in healthy volunteers. J Thromb Haemost 2013; 11(6): 1085-1092; PMID:23530987; http://dx.doi.org/10.1111/jth.12203 23530987

[52] Price PA, Urist MR, Otawara Y. Matrix Gla protein, a new gamma-carboxyglutamic acid-containing protein which is associated with the organic matrix of bone. Biochem Biophys Res Commun 1983; 117(3): 765-71; PMID:6607731; http://dx.doi.org/10.1016/0006-291X(83)91663-7 6607731

[53] Beulens JW, van der A DL, Grobbee DE, Sluijs I, Spijkerman AM, van der Schouw YT. Dietary phylloquinone and menaquinones intakes and risk of type 2 diabetes. Diabetes Care 2010; 33(8):1699-705; PMID:20424220; http://dx.doi.org/10.2337/dc09-2302 20424220 PMC2909045

[54] Liabeuf S, Olivier B, Vemeer C, Theuwissen E, Magdeleyns E, Aubert CE, Brazier M, Mentaverri R, Hartemann A, Massy ZA. Vascular calcification in patients with type 2 diabetes: the involvement of matrix Gla protein. Cardiovasc Diabetol 2014; 13(1): 85; PMID:24762216; http://dx.doi.org/10.1186/1475-2840-13-85 24762216 PMC4017083

[55] Miyake N, Hoshi K, Sano Y, Kikuchi K, Tadano K, Koshihara Y. 1,25-Dihydroxyvitamin D3 promotes vitamin K2 metabolism in human osteoblasts. Osteoporos Int 2001; 12(8): 680-7; PMID:11580082; http://dx.doi.org/10.1007/s001980170068 11580082

